# Impact of irrigation, nitrogen fertilization, and plant density on stay-green and its effects on agronomic traits in maize

**DOI:** 10.3389/fpls.2024.1399072

**Published:** 2024-09-06

**Authors:** Nadia Chibane, Pedro Revilla, Venkata Rami Reddy Yannam, Purificación Marcet, Emma Fernández Covelo, Bernardo Ordás

**Affiliations:** ^1^ Maize Genetics and Breeding Group, Misión Biológica de Galicia [The Spanish National Research Council (CSIC)], Pontevedra, Spain; ^2^ Sustainable Field Crops Program, Institute for Food and Agricultural Research and Technology (IRTA), Lleida, Spain; ^3^ Area de Edafología y Química Agricola, Facultad de Ciencias, Universidad de Vigo, Vigo, Spain; ^4^ Crop Adaptation and Sustainability Group, Misión Biológica de Galicia [The Spanish National Research Council (CSIC)], Pontevedra, Spain

**Keywords:** maize (*Zea mays L*.), leaf senescence, stay-green, abiotic stress, plant density, nitrogen fertilization, water irrigation

## Abstract

**Introduction:**

The stay-green (SG) or delayed leaf senescence enables crop plants to maintain their green leaves and photosynthetic capacity for a longer time after flowering. It is considered an important trait in maize breeding, which has contributed to gain in grain yield of modern varieties. It has been also used to improve the tolerance to drought and deficiencies in nitrogen fertilization (NF). However, the objective of this study is to evaluate the influence of water irrigation (WI), NF, and plant density (PD) on SG and the effect of SG on agronomic traits in maize.

**Methods:**

Four SG lines and four non–stay-green (NSG) lines were evaluated in four contrasting environments under two WI, three NF, and two PD levels.

**Results and discussion:**

As expected, the chlorophyll content of leaves at 45 days after flowering (Chlo45) was, on average, higher in the SG group of lines. The difference in Chlo45 between the SG and NSG genotypes was consistent across WI, NF, and PD and the environments. This is indicative that internal or developmental factors were more important than external signals in controlling the senescence. The effect of SG increasing thousand-kernel weight, stover yield at harvest, or moisture was not influenced by WI, NF, or PD but was altered by the background environment. Our results have implications for the application of SG as a secondary trait for enhancing abiotic stress tolerance. Future studies could consider a wider range of environmental conditions to assess the performance of SG traits under different climatic and soil conditions.

## 1 Introduction

With global climate change and population growth, there is an increasing need for crop yield improvements to ensure food availability and to meet future agricultural production demands. In response to this critical demand, plant breeding must be accelerated to uncover traits that can increase the yield potential and better adapt to abiotic stress. One such strategy is the selection of stay-green (SG) genotypes, which can help meet anticipated population growth demands, particularly under adverse conditions (Harris, 2007; Luche et al., 2015; Kamal et al., 2019). SG genotypes are characterized by delayed senescence and reduced chlorophyll loss compared to non–stay-green (NSG) genotypes (Kamal et al., 2019; Jiao et al., 2020; Wang et al., 2021). This trait is considered important in agriculture as it allows plants to maintain photosynthetic activity, thereby improving the grain-filling process (Clay et al., 2009; Zhang et al., 2019). Maize is one of the three primary cereal crops, ranking third in cultivation, after rice and wheat. It is a versatile plant that can grow in various soils and climates and serves not only as a staple food but also as a raw material for animal feed and bioenergy production. It exhibits a highly efficient C4 photosynthetic mechanism, which results in substantial biomass production (Chen et al., 2015). Studies indicates that delayed senescence in SG maize hybrids can result in increased dry matter accumulation compared to that in NSG hybrids (Pommel et al., 2006). The usefulness of SG extends beyond its positive influence on post-flowering dry matter accumulation and post-flowering nitrogen (N) uptake (PostN) as it also has the capacity to enhance grain yield (Borrell and Hammer, 2000; Ning et al., 2013; Chibane et al., 2021).

Recent maize hybrids exhibit increased dry matter and nitrogen accumulation during grain filling, whereas nitrogen use efficiency (NUE) is inversely correlated with grain nitrogen concentration (GNC) in high-yielding modern hybrids (Rajcan and Tollenaar, 1999; Zhiipao et al., 2023). Studies have revealed that the accumulation of dry matter in maize kernels depends on nitrogen levels. Nitrogen availability is also crucial for determining the allocation, distribution, and reallocation of dry matter and nitrogen in maize (Liu et al., 2023). It has been found that modern maize hybrids with increased nitrogen uptake and partitioning can achieve equilibrium between nitrogen levels prior to silking (Subedi and Ma, 2005). During the post-silking phase, efficient nitrogen uptake is essential to minimize the requirement for nitrogen remobilization from vegetative to reproductive organs. Strategic nitrogen management contributes to the preservation of green leaf area and extension of dry matter (Rajcan and Tollenaar, 1999; Worku et al., 2007). The duration of canopy photosynthesis can be extended by steady nitrogen uptake during grain filling, leading to an increased final grain yield. Modern hybrids exhibit parallel grain yield and nitrogen efficiency due to increased total dry matter at maturity, particularly greater grain dry weight (Mueller et al., 2019). Nitrogen deficiency in maize typically presents visually as reduced leaf area, diminished chlorophyll in mature leaves, and a decrease in the overall vegetation index. These effects lead to a decrease in the capacity of the plant to absorb light and produce photoassimilates, ultimately resulting in lower grain yields (Echarte et al., 2008). Evaluation of maize genotype performance under low-nitrogen conditions has demonstrated substantial differences compared to optimal conditions, with only a small percentage of genotypes showing resilience to low-nitrogen levels (Buchaillot et al., 2019). Recent studies have explored how genotypic variation in maize hybrids affects root anatomy under varying levels of nitrogen stress (Yang et al., 2019). The dynamics of post-silking nitrogen fluxes in maize are essential for grain yield because they affect NUE and the number of kernels. In maize, excessive vegetative nitrogen uptake can help maintain grain yield when there is post-silking nitrogen stress by increasing the number of kernels and remobilizing nitrogen to meet grain nitrogen demand. Post-silking nitrogen deficiency affects carbon partitioning, leading to reduced plant growth and lower grain yield compared to nitrogen-sufficient plants (Ning et al., 2017, 2018; Nasielski et al., 2019). An increase in maize yield over time has been associated with breeding for tolerance to higher plant densities (Antonietta et al., 2014). This suggests that modern maize hybrids have been developed to cope with the challenges posed by higher plant densities, potentially influencing the senescence patterns. The relationship between senescence and maize plant density (PD) is important for understanding crop physiology and optimizing yield (Jia et al., 2018). The greater the density of plants, the fewer the resources available per plant. Higher plant densities can accelerate the rate of leaf senescence, leading to reduced post-silking net photosynthesis and assimilation availability (Borrás et al., 2003; Tong et al., 2019). The reason for this is the elevated level of competition between plants, which leads to increased variability. For instance, dominant plants indulge in excessive nutrient consumption and disadvantaged subordinate plants. This suggests that PD influences the timing, rate, and intensity of senescence, affecting the ability of plants to photosynthesize and allocate resources effectively (Shafi et al., 2012; Burken et al., 2013). The interaction between PD and senescence underscores the complex relationship between resource availability, physiological processes, and crop productivity, a connection that has not been extensively described in the existing literature. In addition to the availability of nitrogen and PD, various levels of water stress can influence senescence and end products of maize in different ways. Mild water deficit conditions have been found to accelerate leaf senescence, which is considered an adaptive response in plants experiencing water shortage (Ye et al., 2020). This acceleration of senescence helps to reduce the overall water demand of the plant during periods of limited water availability (Pic et al., 2002). Under severe water stress conditions, such as post-silking drought, the consequences of leaf photosynthesis and senescence can be substantial and have an impact on grain yield (Trachsel et al., 2016a; Ye et al., 2020). A delay in leaf senescence has a positive influence on yield under water stress and nitrogen conditions, thereby emphasizing the complex interplay between environmental stressors and plant physiological responses (Riache et al., 2023). Furthermore, studies have linked plant senescence characteristics, such as green leaf area, with water availability, suggesting a close relationship between water stress and maize senescence. Overall, water deficit stress during critical growth stages, such as pre-flowering and grain filling, can have a significant impact on maize performance, affecting phenology and yield components owing to altered physiological traits induced by water scarcity (Sah et al., 2020; Wu et al., 2023).

The relationship between SG traits and various physiological and agronomic traits under different stressors has been a topic of interest. SG refers to the ability of crop plants to maintain green leaves and photosynthetic capacity for an extended period, contributing to enhanced drought resistance and performance under low-nitrogen conditions (Thomas and Ougham, 2014; Kamal et al., 2019; Riache et al., 2023). This trait plays a crucial role in delaying foliar senescence, which is essential for sustaining photosynthesis and overall plant productivity (Ramkumar et al., 2019; Munaiz et al., 2020). Studies have demonstrated that the SG phenotype is associated with improved drought tolerance, delayed leaf senescence, and better performance under challenging conditions, such as low nitrogen availability and high PD (Kamal et al., 2019; Munaiz et al., 2020; Riache et al., 2023). In the SG genotypes, there was reduced remobilization of nitrogen from stover to grain, which led to a higher nitrogen content in stover at harvest and a lower content in grain (Zhang et al., 2019). The difference in nitrogen remobilization between the SG and NSG genotypes was less striking than the differences in biomass remobilization. Similarly, other studies have reported higher nitrogen uptake and lower remobilization in SG genotypes after flowering (Thomas et al., 2002; Havé et al., 2017; Zhang et al., 2019). The dynamics of post-silking nitrogen fluxes play a significant role in the NUE and grain yield in maize. It is crucial to understand the balance between nitrogen remobilization from vegetative tissues and post-silking nitrogen uptake to optimize the grain yield under various nitrogen conditions (Ning et al., 2017). Senescence is a complex process involving many biological and genetic influences, in addition to abiotic factors, such as water, nitrogen, and PD, which have been shown to affect various agronomic traits (Sade et al., 2018; Asad et al., 2019). This highlights that the understanding of these interactions between abiotic, nitrogen, and PD stresses for the development of crop improvement strategies remains unclear. The objective of this research is to assess the influence of WI, NF, and PD and environmental background on the progress of senescence and on the effect of SG on agronomic traits of economic interest.

## 2 Materials and methods

### 2.1 Plant materials

Eight inbred maize lines were used in this study, including four SG lines (PHW79, PHW52, PHP38, and PHBW8) and four NSG lines (PHBB3, B73, PHT11, and PHM10) (**Table 1**). These lines were selected from 197 inbred lines evaluated in Misión Biológica de Galicia for senescence-related traits under high water and nitrogen levels (Caicedo, 2018; Chibane et al., 2021). Except for B73, all these lines belong to two heterotic groups that are frequently used as parental breeds (Mikel and Dudley, 2006; White et al., 2020). B73, which belongs to the Stiff Stalk Synthetic (BSSS) heterotic group, a pivotal variety in temperate maize breeding history, has been utilized since the 1970s in developing Stiff Stalk inbreeds and initiating its heterotic components across all hybrids maize breeding programs.

**Table 1:** Stay-green phenotype, heterotic groups, and origin of the eight inbred lines of maize.

Genotypes	Stay-green	Heterotic groups	Origin
**PHBW8**	SG	Amargo (PHG39)	Pioneer ExPVP
**PHW52**	SG	Amargo (PHG39)	Pioneer ExPVP
**B73**	NSG	Stiff stalk	Iowa State University
**PHW79**	SG	Oh07-Midland (PH595)	Pioneer ExPVP
**PHP38**	SG	Amargo (PHG39)	Pioneer ExPVP
**PHT11**	NSG	Amargo (PHG39)	Pioneer ExPVP
**PHM10**	NSG	Amargo (PHG39)	Pioneer ExPVP
**PHBB3**	NSG	Amargo (PHG39)	Pioneer ExPVP

### 2.2 Experimental trial and management practices

The study was conducted in two locations in the Galicia region, Tomeza “TM” (latitude: 42.40°N and longitude: 8.63°W) in Pontevedra province and Xinzo “XZ” (latitude: 42.07N and longitude: 7.73°W) in Ourense province. The experiments were repeated for two years: 2018 and 2019. The experimental design at each location was a split-plot design with four factors: water irrigation (WI), nitrogen fertilization (NF), planting density (PD), and genotypes (G). WI was assigned to main plots that were organized as a completed block design with two replications. NF was nested to WI, PD to NF, and G to PD. WI had two levels: high water level (HW) and low water level (LW). It was irrigated weekly in HW at 25 L/m and with half the amount of water every 15 days in LW at 12.5 L/m. NF had three levels (N1: without NF; N2: NF at 30 kg/ha; and N3: NF at 90 kg/ha). NF was applied two times, prior to sowing with half amount and at V6 stage the last part. N was applied as ammonium nitrate (27%). PD had two levels (a high density of 80,000 plants per ha and a low density of 50,000 plants per ha). Each experimental plot consisted of two rows, each row with 13 double-kernel hills planted manually, each block being 26.6 m × 3.25 m, spacing between rows was 0.8 m and between consecutive hills 0.16 m or 0.25 m for final density of 80,000 and 50,000 plants per ha, respectively. The trials were carried out using standard practices to control weeds and pests at the site [herbicides (pendimethalin 33% and sulcotrione 30%) and insecticides (lambda-cihalotrin 10%)]. Standard fertilization with phosphorus (P_2_O_5_ of 18%, 333 kg/ha) and potassium (potassium sulfate (K_2_O) of 50%, 240 kg/ha) was applied prior to sowing.

### 2.3 Environmental and soil variables

A previous analysis of the nitrogen content in the soil for the first-year trial was performed before sowing. Soil samples from the 0-cm to 30-cm soil layer for each location were collected before planting and analyzed in the laboratory of the University of Vigo. The contents of various nutrients, such as the nitrogen fractions NO_3_^−^, NH_4_^+^, and N, were measured following the method of (Houba et al., 2000) (**Supplementary Table 1**). During the growing seasons of 2018 and 2019 in two locations, meteorological data were downloaded from a regional meteorological service (http://meteogalicia.es), to determine the following variables: average monthly minimum, maximum, and average temperature (Tmin, Tmax, and Tavg, in °C) and precipitation (in L/m) (**Supplementary Figure 1**).

### 2.4 Physiological and phenological data

Chlorophyll content and quantum efficiency of PSII (FvFm) were measured with a portable SPAD recorder (CCM-200, Opti-Sciences, Tyngsboro, MA, USA) and a portable fluorometer (OS-30p, Opti-Sciences, Tyngsboro, MA, USA). Chlorophyll content and FvFm were measured 45 days after flowering (DAF) in the leaves of the principal ear of five plants per plot. Leaves of the principal ear were dark-adapted for 20 min with tweezers collocated in the leaf before measurements of FvFm. Days to silking (SD) and days to anthesis (AD) were recorded as the number of days from planting to the date when 50% of the plants had emerged silks and shed pollen, respectively. Then, we estimated the anthesis silking interval (ASI) as the difference between SD and AD. We considered that a plot had reached physiological maturity when at least five ears had a black layer on the seed basis and calculated the number of days from flowering to the physiological maturity of each plot (DPM).

### 2.5 Agronomic variables

The harvest was done after physiological maturity and dry down of the grain at the end of the cultivation season. Stover (leaves and stems) moisture was calculated as the difference between the fresh and dry weights of five random plants in each plot at flowering (SMF) and harvest (SMH). Similarly, kernel moisture [KM (%)] and cob moisture [CM (%)] were calculated as the difference between fresh and dried grains using five random ears of each plot at harvest. Ten plants were collected randomly from each plot at harvest to estimated thousand kernel weight [TKW (g)], cob yield [CY (kg/ha)], and stover yield at harvest SYH (kg/ha). Similarly, stover yield was taken at flowering SYF (kg/ha) sampling 10 plants per plot. All weights and yields were corrected by their respective moistures and were given at 0% of moisture.

### 2.6 Nitrogen content

Total nitrogen content [TN_HSoil (g/kg)] and nitrogen assimilable by plants [NO_3__HSoil (mg/kg) and NH_4__HSoil (mg/kg)] were calculated in soil samples taken from plots of six genotypes (three SG and three NSG) in the first year of the experiment at both locations at harvest. Total nitrogen and nitrogen assimilable by plants in soil were analyzed using elemental analysis (Flash EAI112 series) (Krotz and Giazzi, 2000). In the plots in which soil samples were taken, five random plants were collected at flowering and harvest, and the total nitrogen concentration was measured in the plant stover at flowering SNF (g/kg) and harvest SNH (g/kg) and in the kernels KN (g/kg) using elemental analysis (Flash EAI112 series).

### 2.7 Statistical analyses

Individual and combined analyses of variance (ANOVA) was performed for both years and locations using the mixed-model procedure (MIXED procedure) of the SAS statistical package (https://odamid-euw1.oda.sas.com/SASStudio). WI, NF, PD, and SG were considered fixed effects, and environment and blocks (environment) were considered random effects. Each environment was represented by one location in a year. Interaction terms only involving fixed effects were considered fixed, and interactions terms with at least one factor random were considered random. After doing the analyses, we found that the interactions were mostly not significant, and we repeated the analyses without including the interactions in the model. The Wald test was used to test if the variances were significantly different from cero (Covtest option of mixed procedure of SAS). Comparisons between means were made using Fisher’s protected least significant difference (MDS) test at 5% probability. Combined and individual correlation analyses were performed using R Studio (R Core Team, 2013) with the sjPlot package (Lüdecke, 2021). The correlations between variables were assessed using Pearson’s correlation coefficients with their significance.

## 3 Results

### 3.1 Correlation between SG phenotype and agronomic traits

**Figure 1:**
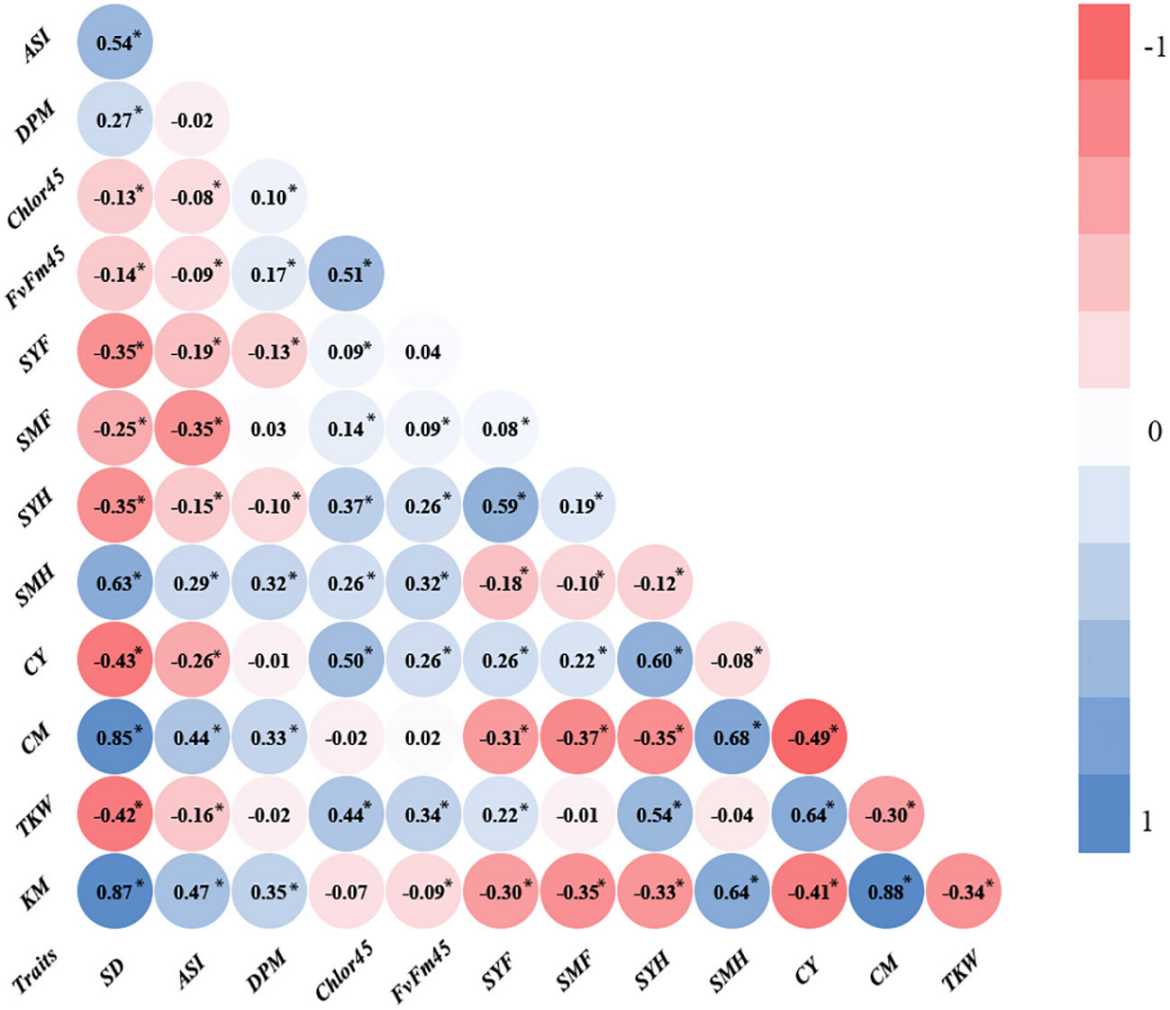
Pearson correlation coefficients between traits estimated from all plots of the experiment. SD, days to silking (days); ASI, anthesis silking interval (days); DPM, days to physiological maturity (days); Chlor45, chlorophyll at 45 days after silking; FvFm45, quantum efficiency at 45 days after silking (μmol m^−2^ s^−1^); SYF, stover yield at flowering (kg/ha); SMF, stover moisture at flowering (%); SYH, stover yield at harvest (kg/ha); SMH, stover moisture at harvest (%); CY, cob yield (kg/ha); CM, cob moisture (%); TKW, thousand kernel weight (g); KM, kernel moisture (%). *, significant at P ≤ 0.05.

**Figure 2:**
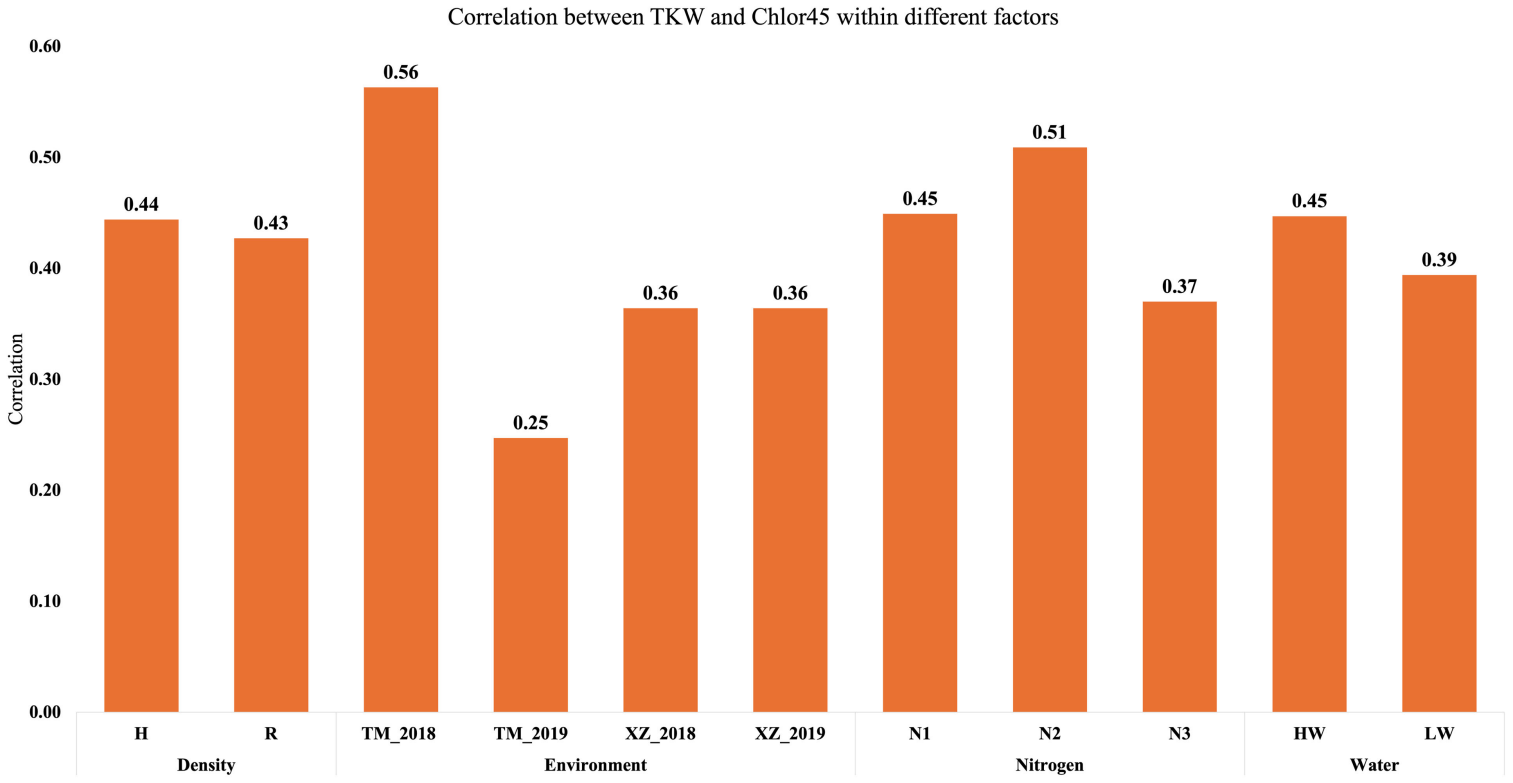
Correlation analysis between Chlor45 (SPAD) and TKW (g) within different factors and environments (HW, high water irrigation; LW, low water irrigation; N1, N2, and N3, different nitrogen fertilization levels; H, high plant density; R, reduced plant density).

The main objective of this research was to analyze the relationship of senescence or SG with agronomic traits and abiotic factors, specifically, WI, N fertilization, and PD. In our environmental conditions, the differences between SG and NSG genotypes are usually highest at 45 DAF (Chibane et al., 2021). For that reason, we used Chlor45 and FvFm45 to analyze the relationship of senescence with agronomic traits and genetic and abiotic factors. In addition to those variables related to leaf senescence, we measured variables related to phenology (flowering and grain filling) and variables related to yield and moisture of grain, stover, and cob. Regarding grain yield, we measured the yield component (TKW) that is more directly related to senescence (Chibane et al., 2021). The simple correlation between traits estimated from all plots of the experiment has shown that Chlor45 has moderate positive relationship (0.4 < r < 0.5, significant) with the weight of the kernels and the yield of stover (at harvest) and cobs (**Figure 1**). The correlation between Chlor45 and SMH was slightly lower (r = 0.26) and significant. However, there was not relationship between Chlor45 and the moisture of kernels and cobs (0.07 and −0.02, respectively, not significant). The correlations of FvFm45 with agronomic traits were similar to Chlor45, although the magnitude of the correlations was mostly lower. The relationships of other phenological traits with agronomic traits were different from those found with Chlor45 (**Figure 1**). Thus, SD had high or very high positive relationships with moisture related traits (0.87 for KM and 0.85 for CM, significant) but moderate negative with yield related traits. DPM had also positive relationship with moisture related traits but not of high magnitude and did not have correlations with yield related traits (**Figure 1**). The only physiological traits that had positive correlations with TKW and CY were Chlor45 and FvFm45. The correlation between Chlor45 and TKW within each level of water, N, and PD was similar to the combined value (0.4 < r < 0.5, significant), that is, the correlation between the two traits was not affected by those factors (**Figure 2**). If we considered the correlations between the two traits within environments, then we found similar values to the combined value in three of the environments, but, in Tomeza 2019, the correlation was slightly lower (**Figure 2**).

### 3.2 Comparison of SG vs. NG lines: main effects

The analysis of correlations suggests two main results: the phenological trait with highest effect on kernel weight is senescence or SG, and this effect is independent of WI, N fertilization, and density. However, the specific conditions of the environments across the season could have an impact on the relationship of SG and kernel weight. To further investigate the effect of SG on agronomic traits and how the environment and WI, N fertilization, and density influence SG and alter the effect of SG on agronomic traits, we compare a group of four SG genotypes and a group of four NSG genotypes evaluated in four environments under different levels of the abiotic factors using an ANOVA model. The interactions between abiotic factors, SG, and environment were mostly not significant (data not shown). The reduced model without interactions has shown that the differences between the SG and NSG groups (SG factor) were significant (α ≤ 0.05) for several phenological and agronomic traits (**Table 2**). The SG group had higher values of DPM, Chlor45, FvFm45, and higher yield and moisture of stover at harvest, cobs, and kernels (SYH, CY, TKW, SMH, CM, and KM) (**Supplementary Tables 2–4**).

**Table 2:** Test of fixed effects for the mixed-model ANOVA combined over environments.

Trait	Effect	WI	NF	PD	SGT	ENV
SD	F-value	0.34	3.66	10.01	1.00	122.08
	Pr > F	0.57	0.03	0.00	0.32	<0.0001
ASI	F-value	2.19	3.20	7.09	1.35	14.07
	Pr > F	0.17	0.05	0.01	0.25	0.00
DPM	F-value	2.39	0.85	0.27	33.77	14.69
	Pr>F	0.15	0.43	0.60	<0.0001	0.00
Chlor45	F-value	8.87	15.01	2.52	95.89	9.67
	Pr > F	0.01	<0.0001	0.12	<0.0001	0.00
FvFm45	F-value	3.49	0.67	0.08	11.55	2.93
	Pr > F	0.09	0.51	0.78	0.00	0.08
SYF	F-value	0.58	0.65	156.01	11.67	9.03
	Pr > F	0.47	0.52	<0.0001	0.00	0.03
SMF	F-value	12.20	11.13	2.10	104.86	30.81
	Pr > F	0.01	<0.0001	0.15	<0.0001	<0.0001
SYH	F-value	13.59	3.83	153.40	9.74	27.88
	Pr > F	0.01	0.03	<0.0001	0.00	0.00
SMH	F-value	0.71	0.40	0.17	31.93	37.73
	Pr>F	0.42	0.67	0.81	<0.0001	<0.0001
TKW	F-value	12.54	2.24	4.67	215.99	69.18
	Pr > F	0.00	0.11	0.03	<0.0001	<0.0001
KM	F-value	0.50	0.48	0.61	12.28	7.26
	Pr > F	0.50	0.62	0.43	0.00	<0.0001
KN	F-value	3.08	0.03	0.39	8.36	15.71
	Pr > F	0.12	0.97	0.54	0.00	0.01
CY	F-value	14.31	3.89	121.68	136.81	54.93
	Pr > F	0.00	0.03	<0.0001	<0.0001	<0.0001
CM	F-value	0.00	3.73	5.96	13.16	97.66
	Pr > F	0.97	0.02	0.01	0.00	<0.0001
SNH	F-value	1.44	8.91	0.33	15.45	4.93
	Pr > F	0.26	0.00	0.57	<0.0001	0.02
SNF	F-value	0.32	20.56	2.54	0.04	5.21
	Pr > F	0.59	<0.0001	0.12	0.84	0.01
SNH	F-value	1.39	9.05	0.48	16.09	4.95
	Pr > F	0.26	0.00	0.49	<0.0001	0.02
TN_Hsoil	F-value	0.09	0.56	0.00	2.46	0.02
	Pr > F	0.76	0.57	0.98	0.11	0.89
NO_3__Hsoil	F-value	2.41	2.41	0.04	0.00	6.42
	Pr > F	0.18	0.11	0.84	0.97	0.05
NH_4__Hsoil	F-value	0.85	0.99	0.01	0.64	0.05
	Pr >F	0.36	0.38	0.91	0.42	0.85

WI, water condition; NL, nitrogen level; PD, plant density; SGT, stay-green trait; ENV, environment; DS, days to silking; ASI, anthesis silking interval; DPM, days to physiological maturity. Chlo45 (SPAD), chlorophyll at 45 days after silking; FvFm45 (μmol m^−2^ s^−1^), quantum efficiency at 45 days after silking; SYF(kg/ha), stover yield at flowering (kg/ha); SMF, stover moisture at flowering (%); SYH (kg/ha), stover yield at harvest time (kg/ha); SMH, stover moisture at harvest (%); TKW (g), thousand kernel weight; KM (%), kernel moisture; KN (g kg^−1^), N-kernel content at harvest time; CY, cob yield (kg/ha); CM, cob moisture (%); SNH, stover N at harvest (g kg^−1^); SNF, stover N content at flowering time (g kg^−1^); SNH, stover N at harvest (g kg^−1^); TN_Hsoil (g kg^−1^), total N content in soil at harvest time; NO_3__Hsoil (mg kg^−1^), soil content of NO_3_ at harvest time; NH_4__Hsoil (mg kg^−1^), soil content of NH4 at harvest time.

#### 3.2.1 Stover yield

Not only SG but also WI, N fertilization, and PD had significant effects on SYH (**Table 2**). The magnitude of the effect due to WI was almost twice than SG (**Supplementary Table 3**). High PD, which means fewer resources, reduced the SYH measured on a plant basis, but not when measured per hectare, because a higher number of plants compensated for less production per plant (**Supplementary Table 3**). The stover yield at flowering (SYF) averaged over all plots of the experiment was reduced at harvest (SYH). This is indicative of the translocation of organic matter from the leaves and stems to the kernels during grain filling. Higher WI, higher N fertilization, and lower PD (measured on plant basis) incremented the stover yield at flowering (**Supplementary Table 3**). The differences were significant or close to significant levels. This suggests that the effects of those factors on stover yield started before flowering. The differences in remobilization from stover to grain (SYF/SYH × 100) among the levels of these factors were low. Therefore, the differences on stover yield at harvest due to those factors were probably generated mostly by differences in the availability of resources for the plant that started before flowering, rather than to differences in remobilization. In contrast, the NSG lines even had more stover at flowering than the SG lines (**Supplementary Table 3**), but they had 12% more remobilization. Therefore, at difference of the abiotic factors, the difference in SYH between SG and NSG was mostly due to the differences in remobilization.

#### 3.2.2 Kernel weight and moisture

**Figure 3:**
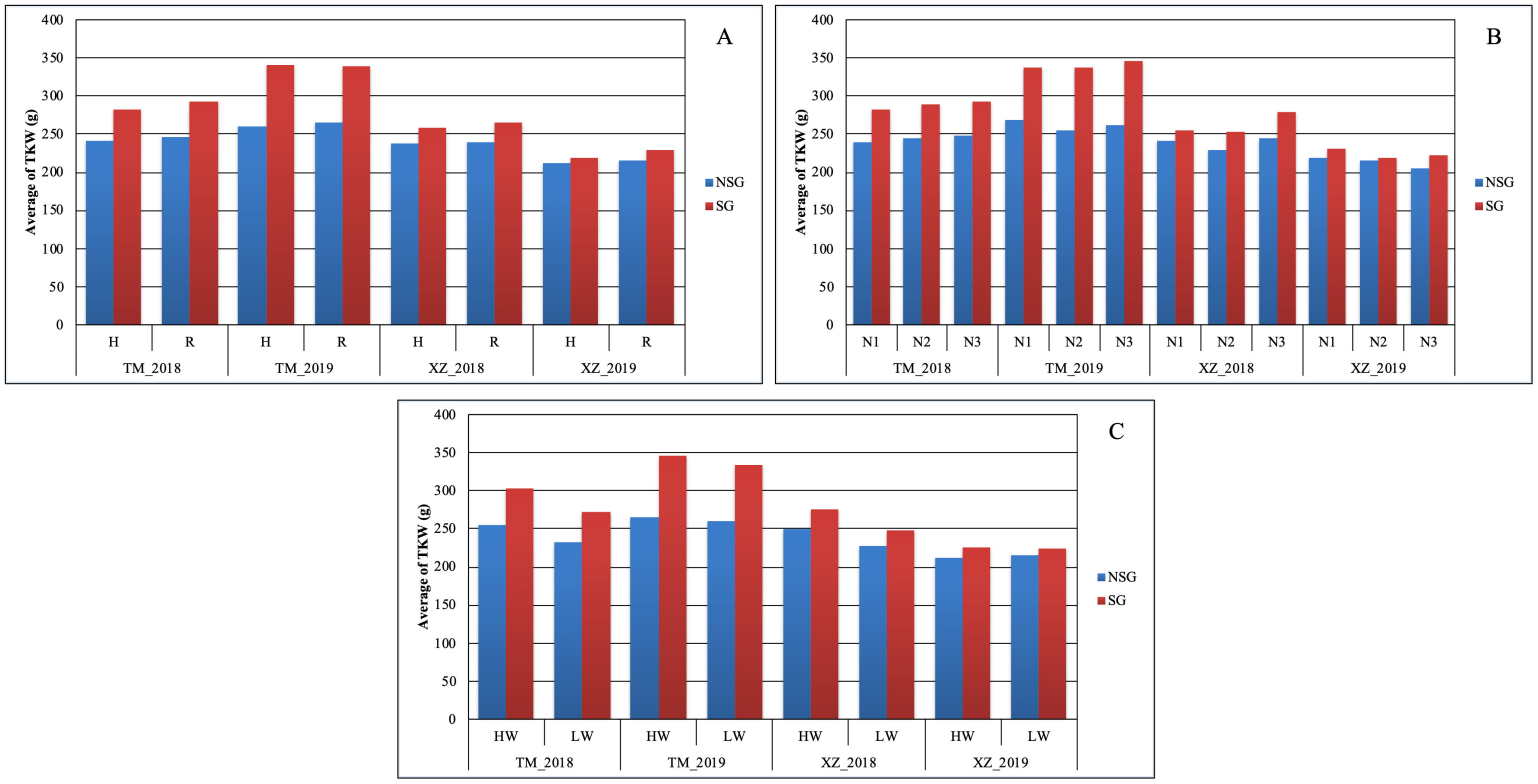
Average of thousand kernel weight TKW (g) within each abiotic stress of **(A)** plant density, **(B)** nitrogen fertilization, and **(C)** water irrigation for SG and NSG genotypes (HW, high water irrigation; LW, low water irrigation; N1, N2, and N3, different nitrogen fertilization levels; H, high plant density; R, reduced plant density).

At difference of SYH, the effect of SG on TKW was higher than the abiotic factors. The only abiotic factor with significant effect on this trait was WI, but the magnitude of the effect of SG on TKW was about twice of the WI (39.3g vs. 14.5g) (**Figure 3**). The advantage of SG on TKW comes to the cost of a higher KM: SG was the only factor in which significant differences were detected for this variable (**Table 2**; **Figure 3**). The SG influenced also more than the other factors the moisture of the stover at harvest and increased the moisture of the cob, although in this case the magnitude was similar to other factors (**Supplementary Tables 3, 4**). The increment of kernel weight and moisture in SG genotypes can be partially due to the increment in days from flowering to physiological maturity as DPM was affected by SG more than by other factors (**Supplementary Table 2**).

### 3.3 Interaction of SG with abiotic factors and environments

**Figure 4:**
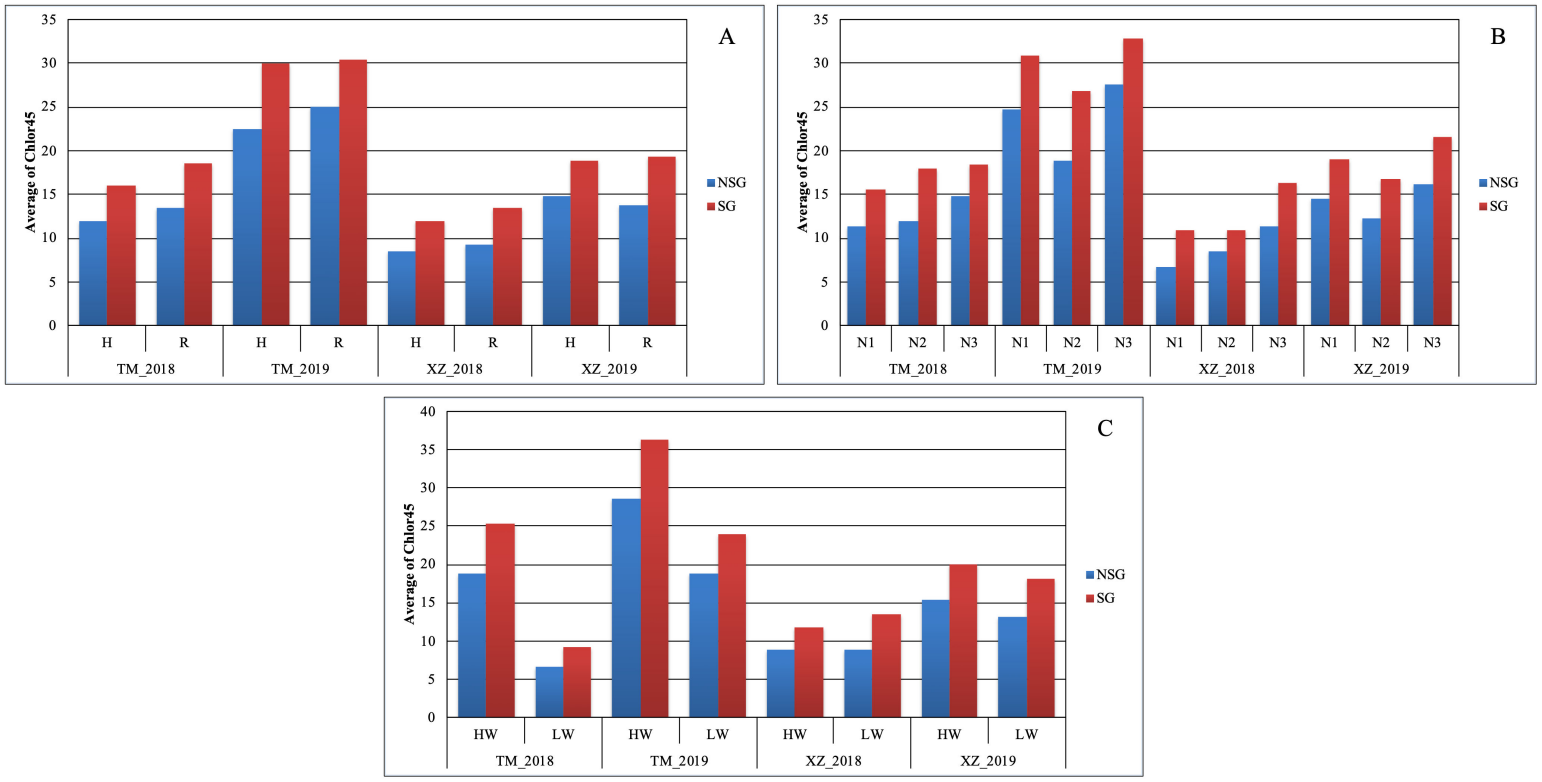
Average of chlorophyll content at 45 days after silking (Chlor45) within each abiotic stress of **(A)** plant density, **(B)** nitrogen fertilization, and **(C)** water irrigation for SG and NSG genotypes (HW, high water irrigation; LW, low water irrigation; N1, N2, and N3, different nitrogen fertilization levels; H, high plant density; R, reduced plant density).

**Figure 5:**
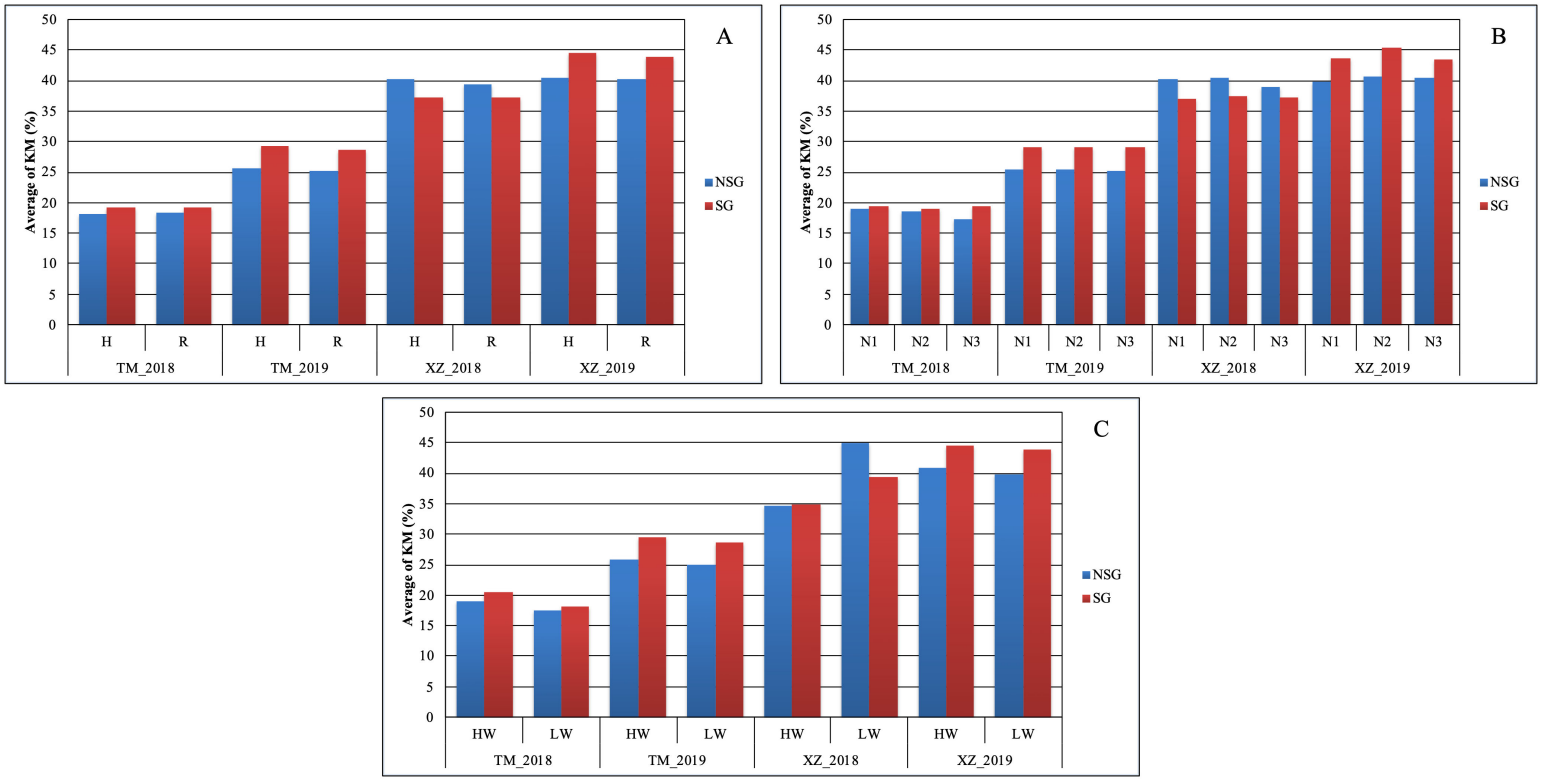
Average of kernel moisture (KM %) within each abiotic stresses of **(A)** plant density, **(B)** nitrogen fertilization, and **(C)** water irrigation for SG and NSG genotypes (HW, high water irrigation; LW, low water irrigation; N1, N2, and N3, different nitrogen fertilization levels; H, high plant density; R, reduced plant density).

In agreement with the lack of significance between SG and environmental and abiotic factors in the full model ANOVA (data not shown), the SG group had higher Chlor45 and TKW than the NSG group across specific abiotic factors and across environments (**Figures 3**, **4**). The magnitude of the difference between SG and NSG for Chlor45 and TKW was similar for the different levels of WI, N fertilization, and density within each environment, but the magnitude varied between environments. There were significant differences between environments for most of the traits (**Table 2**). In Tomeza, the minimum temperatures were higher than in Xinzo along the crop season (**Supplementary Figure 1**). The lower temperatures that imply lower growing degrees days contributed to later DS and also to shorter DPM, lower SYF and TKW, and higher KM in Xinzo compared to Tomeza (**Table 3**). The differences in production between locations were more pronounced in 2019, which had the best (Tomeza 2019) and worst (Xinzo 2019) environment of the experiment. In Tomeza 2019, the average chlorophyll content was still high at 45 days (Chlor45 = 27), indicating a low progress of senescence and a longer period of active photosynthesis compared to Xinzo 2019 (Chlo45 = 18). The difference between SG and NSG on Chlor45 and particularly TKW tended to be higher as the environment was more productive. In other traits related to biomass production (SYH and CY), the SG lines were consistent across factors and environments, superior to NSG lines (**Supplementary Figures 2, 4**). For these traits, the magnitude of the difference between SG and NSG tended to be also higher in the more productive environments, particularly Tomeza 2019. The SG lines tended to have higher KM than NSG lines; however, the magnitude of the difference and the consistency across levels of abiotic factors and environments were lower than for yield related traits (**Figure 5**). Even in Xinzo 2018, the difference between SG and NSG lines for KM had the opposite sign to that in the other environments. The magnitude and consistency across factors and environments of the difference of SG and NSG lines for other moisture related traits (SMH and CM) were similar to those of KM (**Supplementary Figures 3, 5**).

**Table 3:** Mean and standard deviation of traits across different environments.

Env	DS Mean ± SD	DPM Mean ± SD	SYF Mean ± SD	TKW Mean ± SD	KM Mean ± SD
TM_2018	76.69 ± 1.24	76.19 ± 2.47	8,209.68 ± 2,446.14	265.81 ± 28.18	18.8 ± 1.42
TM_2019	81.49 ± 0.94	75.74 ± 3.94	8,542.91 ± 1,654.41	301.22 ± 40.96	27.23 ± 1.97
XZ_2018	88.48 ± 2.07	80.04 ± 2.68	6,541.52 ± 1,746.5	250.46 ± 24.08	38.54 ± 4.81
XZ_2019	96.4 ± 1.76	84.2 ± 2.34	5,788.09 ± 1,333.6	219.08 ± 11.84	42.26 ± 2.41

ENV, environments; SD, standard deviations; DS, days to silking (days); DPM, days to physiological maturity; SYF (kg/ha), stover yield at flowering (kg/ha); TKW (g), thousand kernel weight; KM (%), kernel moisture.

### 3.4 Nitrogen assimilation and remobilization

**Figure 6:**
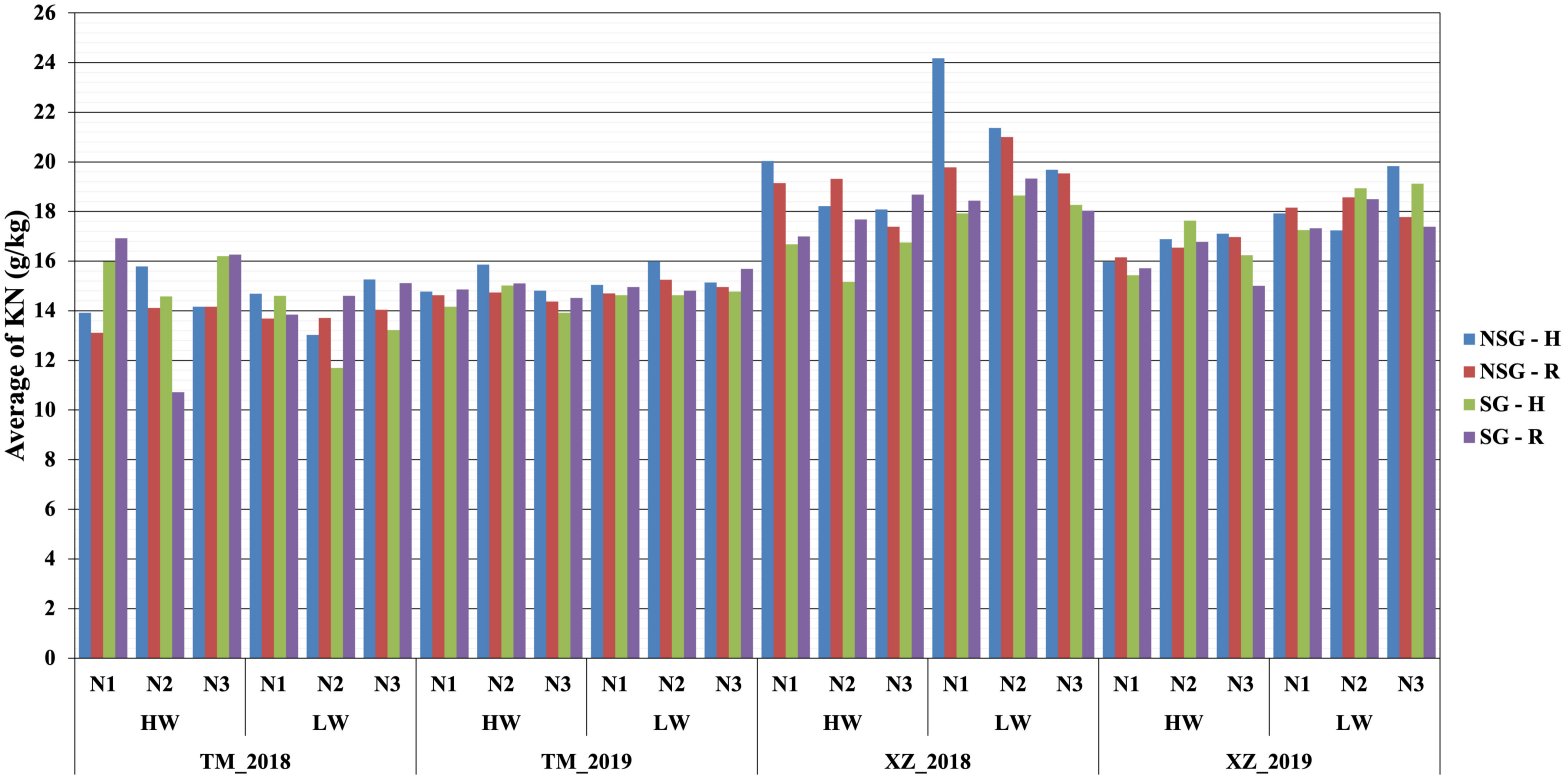
Average nitrogen kernel content KN (g/kg) within combined abiotic stresses of water, nitrogen, and high plant density for SG and NSG genotypes (HW, high water irrigation; LW, low water irrigation; N1, N2, and N3, different nitrogen fertilization levels; H, high plant density; R, reduced plant density).

**Figure 7:**
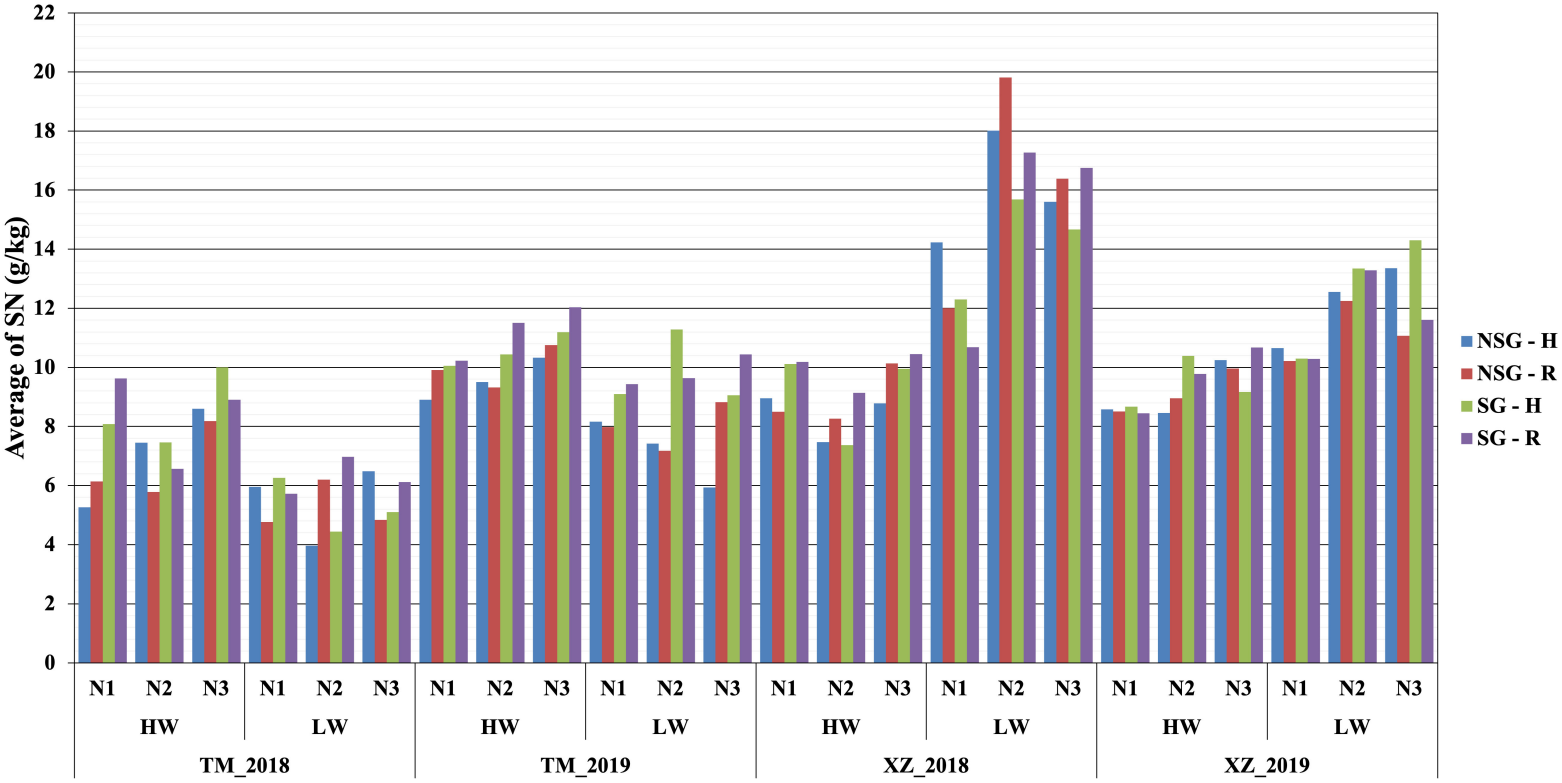
Average of nitrogen stover at harvest SNH (g/kg) within combined abiotic stresses of water, nitrogen, and high plant density for SG and NSG genotypes (HW, high water irrigation; LW, low water irrigation; N1, N2, and N3, different nitrogen fertilization levels; H, high plant density; R, reduced plant density).

At flowering, the only factor that affected the nitrogen concentration in plants was NF with the highest level of N fertilization having significantly more N concentration in plants than the other two levels (**Table 2**; **Supplementary Table 4**). At harvest, the N fertilization also influenced the N concentration in the stover but not in the kernels. The SG lines had higher amount of no remobilized N that remained in the stover at harvest. The concentration of N in the kernels was lower in the SG genotypes, but, taking into account the larger weight of the kernels, the total content of N in the kernels was not reduced compared to that in NSG genotypes. The effect of WI was different to N fertilization as there were not significant differences between levels of irrigation on the concentration of N on stover and grain at harvest. According to the ANOVA analyses, for plant N concentration–related traits, most of the interactions between factors were not significant (data not shown). However, the differences between SG and NSG were not consistent across levels of factors and environments (**Figures 6**, **7**). The reduced ANOVA did not detect significant differences between SG and between levels of abiotic factor for the concentration of N in the soil (**Table 2**).

## 4 Discussion

### 4.1 Senescence or stay-green: main effects

Considering the general average effect across environments and abiotic factors, the SG genotypes had higher biomass yield at harvest in agreement with different studies (Borrell et al., 2001; Pommel et al., 2006; Christopher et al., 2014). In all levels of factors, there were reduction of biomass from flowering to harvest, indicative of the remobilization of biomass and nutrients from the vegetative parts (leaves and stalks) to the grains (**Supplementary Table 3**) (He et al., 2004; Ning et al., 2013). Given that the SG genotypes did not produce higher stover yield at flowering, we inferred that the higher stover yield at harvest of the SG genotypes compared to that of NSG genotypes was mainly due to less remobilization of the biomass generated before flowering. The difference in remobilization between SG and NSG (12%) was twice or higher than the differences between the levels of the other factors in this study (**Supplementary Table 3**). SG genotypes had also, on average, more kernel weight across factors and environments (**Figure 3**) accordingly to studies of Silva et al. (2003) and Chibane et al. (2021). A comparison of old and new hybrids has shown that SG has contributed to genetic gain in grain yield over the last decades (Pommel et al., 2006; Antonietta et al., 2014), and it is considered an important trait in maize breeding (Gregersen et al., 2008; Gnädinger, 2018; Chibane et al., 2021). Despite the lower remobilization, the weight of the kernels was higher, indicating more post-flowering generation of assimilates and nutrient uptake favored by the elongation of the period with active photosynthesis in leaves and the period of filling in the grain. Other authors have also found higher post flowering uptake and lower remobilization in SG genotypes (Rajcan and Tollenaar, 1999; Kosgey et al., 2013; Acciaresi et al., 2014; Chibane et al., 2021), which was named “dilemma of senescence” (Bänziger et al., 2000; Wang et al., 2016). Consistently with the biomass remobilization, in the SG genotypes, there was less remobilization of N from stover to grain (**Supplementary Table 4**), which caused more N content in stover at harvest. This effect is more evident if we consider the absolute value of N content (N per ha) instead of g per Kg because the SG genotypes had higher stover yield at harvest (**Supplementary Table 4**). The SG genotypes had less N content in grain in percentage or g per kg of the grain (**Supplementary Table 4**), but, if we adjust for the higher TKW of the SG genotypes, assuming that SG and NG genotypes have the same number of kernels, we found that the absolute N content in grain was also higher in SG genotypes. This is indicative of more N uptake after flowering in SG genotypes (Subedi and Ma, 2005). The average favorable effect of SG on yield of stover, cob, and yield was accompanied by an increment in the moisture (**Figures 3**, **5**; **Supplementary Tables 3, 4**). The trade-off between grain yield and moisture associated with senescence (similar to flowering time) is sometimes neglected: a higher grain yield could not be of value if it was accompanied by excessive moisture. In fact, it is not rational to consider that SG is better than NSG or vice versa but to consider the timing and rate of senescence as relevant traits for adaptation that should be optimized for the specific environment in which the genotypes are going to grow (Munaiz et al., 2020). For the study of senescence to be useful in breeding, it is necessary to integrate it with genotypic and environmental factors, as well as other characteristics that determine the final performance in the target environment. Trachsel et al. (2016b) proposed that reducing flowering time and lengthening senescence, which maintains the duration of the crop, does not increase humidity and increases early vigor to produce greater vegetative biomass that compensates for the shorter time until flowering.

### 4.2 Senescence or stay-green: interactions with environmental factors

We found large difference between environments, for example, for TKW or KM, indicating that the environments were quite contrasting (**Figures 3**, **5**). The lower levels of the abiotic factors consistently caused ASI lengthening, which is indicative of stressful conditions for the maize plants (Bänziger et al., 2000) (**Supplementary Table 2**), and appreciable differences among levels of factors were achieved in this experiment. In spite of those differences, the difference in Chlor45 between the SG and NSG genotypes were consistent across environments and levels of abiotic factors (**Figure 4**). This trait serves to compare the progress of senescence and our data support the notion that the progress of senescence, including timing and progress, is controlled mainly by internal factors in maize, for example, hormone accumulation, which is relatively independent of external signals (Borrás et al., 2003), also found that the progress of senescence is highly conservative and concluded that it is genetically controlled. Differences in senescence had an effect on traits of agronomic relevance, particularly TKW. An interesting question is how extrinsic factors, such as environmental conditions or other biotic factors modify the effects of the differences of senescence on agronomics traits. The abiotic factors that did not alter the progress of senescence did not alter the effect of senescence on traits like TKW or KM (**Figures 3**, **5**). However, the background environment altered the effect of senescence on agronomic traits. Our data suggest that, in the environments that are more productive and where the lines are better adapted, the effect of senescence on agronomic trait is more pronounced. Growing degree days and the total duration of the cultivation cycle are the factors that probably contributed to the effects of the background environments. Analyses of more environments and detailed environmental characterization of them could provide relevant information about the environmental determinants of the effects of the senescence. The relationship between senescence and different factors can be inferred by the analysis of the relative strength of the sources (duration of photosynthesis activity, availability of water and N, etc.) and sinks (characteristics of the ear and grains, etc). However, the analyses of the relationship of sources and sinks in relation to senescence provided contradictory results; for example, reducing the sink strength by ear removal or prevention of pollination could accelerate senescence (Rajcan and Tollenaar, 1999) or delay it (Borrás et al., 2003). In a thoughtful analysis of this question, Abeledo et al. (2020), altering the source and sink ratio by pollination prevention, ear removal, and partial defoliation, found that there is no consistent effect of specific environmental changes on the source–sink ratio on senescence. However, similar to other study, the authors found that the response of senescence to changes in the source–sink ratio depends on the background environment. Related to this subject, Martin et al. (2005) raised the interesting question of whether remobilization in leaves started because of the progress of senescence or an increased demand and remobilization of nutrients from leaves triggers the onset of senescence. However, our present and previous research did not allow to resolve this question, and specific designs are needed to answer it. Independent of the internal mechanism of the senescence, the fact that the effect of the senescence on agronomic traits is not altered by abiotic stresses raises doubts about the usefulness of SG as a secondary trait for improving abiotic stress tolerance (Kumar et al., 2022; Ali et al., 2023; Riache et al., 2023; Santos et al., 2023).

### 4.3 Abiotic factors

Similarly to SG, higher WI resulted in less biomass remobilized to the grains that were, in spite of that, heavier (**Supplementary Figures 2, 3**). However, at difference of SG, there was not significant difference between levels of irrigation on the concentration of N in the stover at harvest. Corrected by the stover yield, the magnitude of the total content of N in stover at harvest is higher in the low WI level, which suggests that high WI favors N remobilization. There was no difference in the N concentration between levels of irrigation, but, given that the kernels were heavier with high irrigation, the total N content in kernels was not lower or could be even higher if the higher irrigation would have favored a higher number of kernels (Guo et al., 2023; Liu et al., 2024) (**Figures 6**, **7**; **Supplementary Table 4**). The main effect of N was quite different to SG and WI, as affected only the yield and N concentration of stover but did not have effect on grains.

## 5 Conclusions

The progress of senescence is very stable across levels of abiotic factors (water, nitrogen, and density) and background environments, supporting the hypothesis that senescence is primarily controlled by internal factors in maize and remains relatively independent of external signals. The effect of stay green on agronomic traits, particularly TKW, is not affected by abiotic factors but is affected by the background environment. Our results have implications for the application of SG as a secondary trait for enhancing abiotic stress tolerance. Future studies could consider a wider range of environmental conditions to assess the performance of SG traits under different climatic and soil conditions.

## Data Availability

The original contributions presented in the study are included in the article/**Supplementary Material**. Further inquiries can be directed to the corresponding author.
