# Supplementary material for: Impact of irrigation, nitrogen fertilization, and plant density on stay-green and its effects on agronomic traits in maize
Source: Front Plant Sci. 2024 Sep 6;15:1399072. doi: 10.3389/fpls.2024.1399072 (PMC11414411; doi:10.3389/fpls.2024.1399072)
Supplement: Supplementary file 1 [file DataSheet1.docx]

Supplementary Figure1: Temperature and precipitation data during both growing season 2018 and 2019 in both locations (Tomeza and Xinzo).


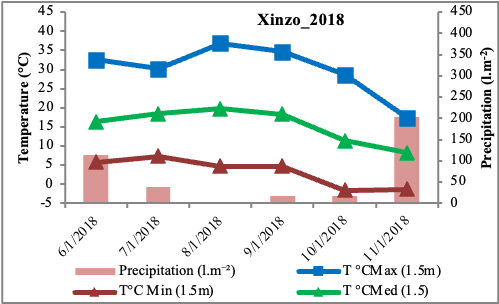

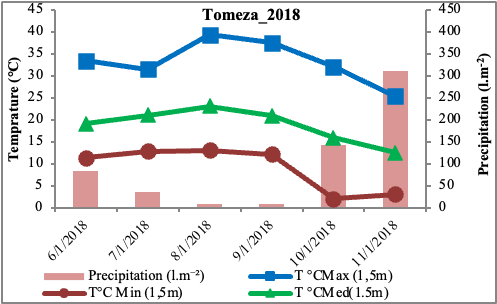

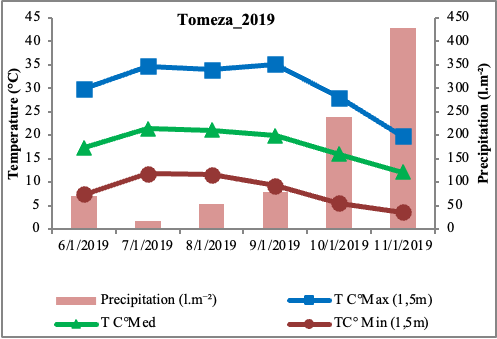

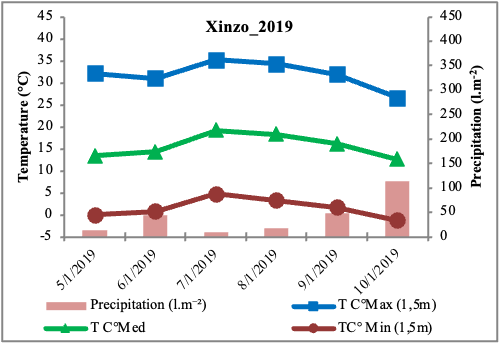


**
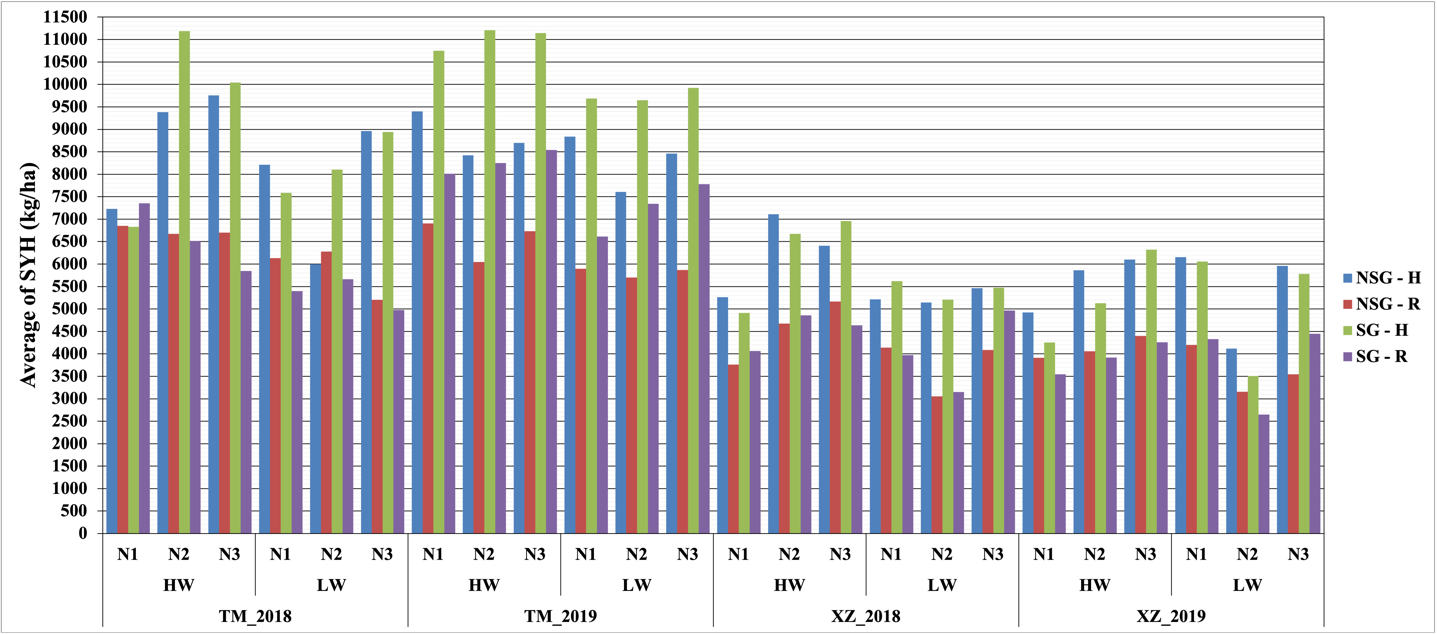
**

**Supplementary Figure 2** Stover yield at harvest SYH (kg/ha) within different stress of water, nitrogen and high plant density for SG and NSG genotypes. (HW: high water irrigation, LW: low water irrigation, N1, N2 and N3: different nitrogen fertilization levels, H: High plant density, R: reduced plant density), Environments (TM_2018, TM_2019, XZ_2018, XZ_2019).

**
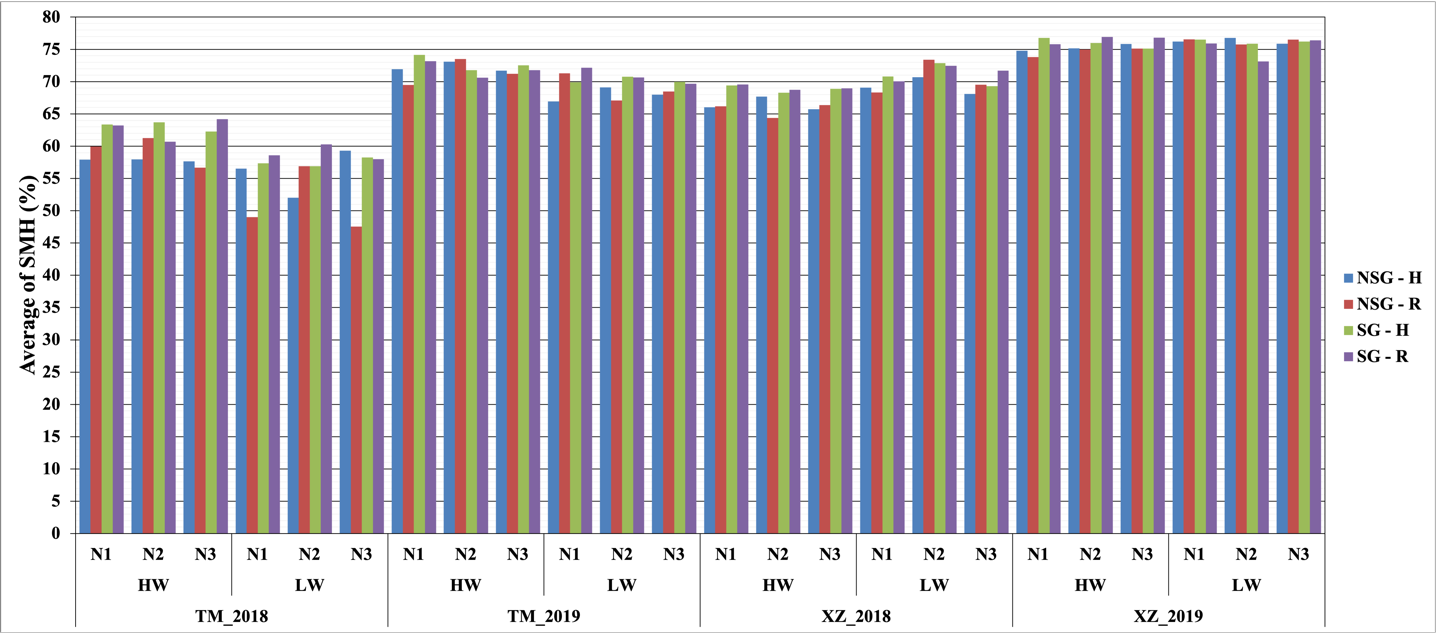
**

**Supplementary Figure 3.** Average of stover moisture at harvest SMH (%) within abiotic stresses of water, nitrogen and high plant density for SG and NSG genotypes. (HW: high water irrigation, LW: low water irrigation, N1, N2 and N3: different nitrogen fertilization levels, H: High plant density, R: reduced plant density) Environments (TM_2018, TM_2019, XZ_2018, XZ_2019).


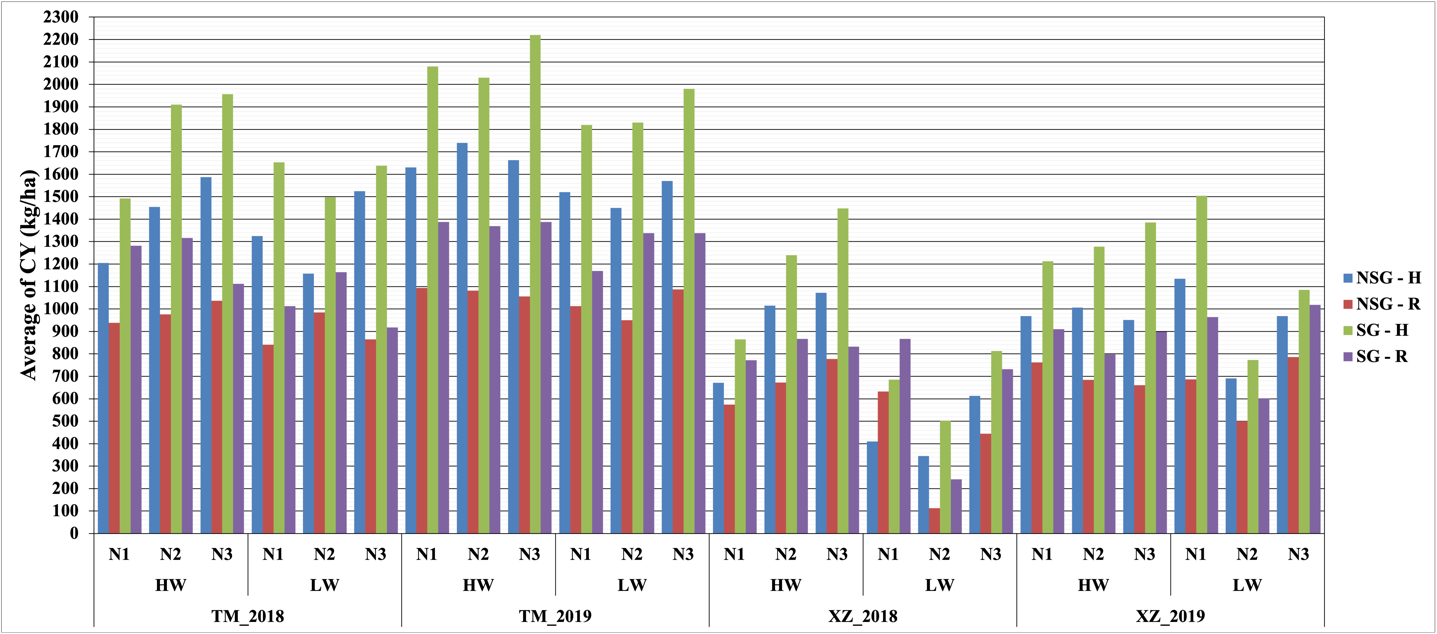


**Supplementary Figure 4.** Average of cob yield CY (Kg/ha) within abiotic stresses of water, nitrogen and high plant density for SG and NSG genotypes. (HW: high water irrigation, LW: low water irrigation, N1, N2 and N3: different nitrogen fertilization levels, H: High plant density, R: reduced plant density). Environments (TM_2018, TM_2019, XZ_2018, XZ_2019).


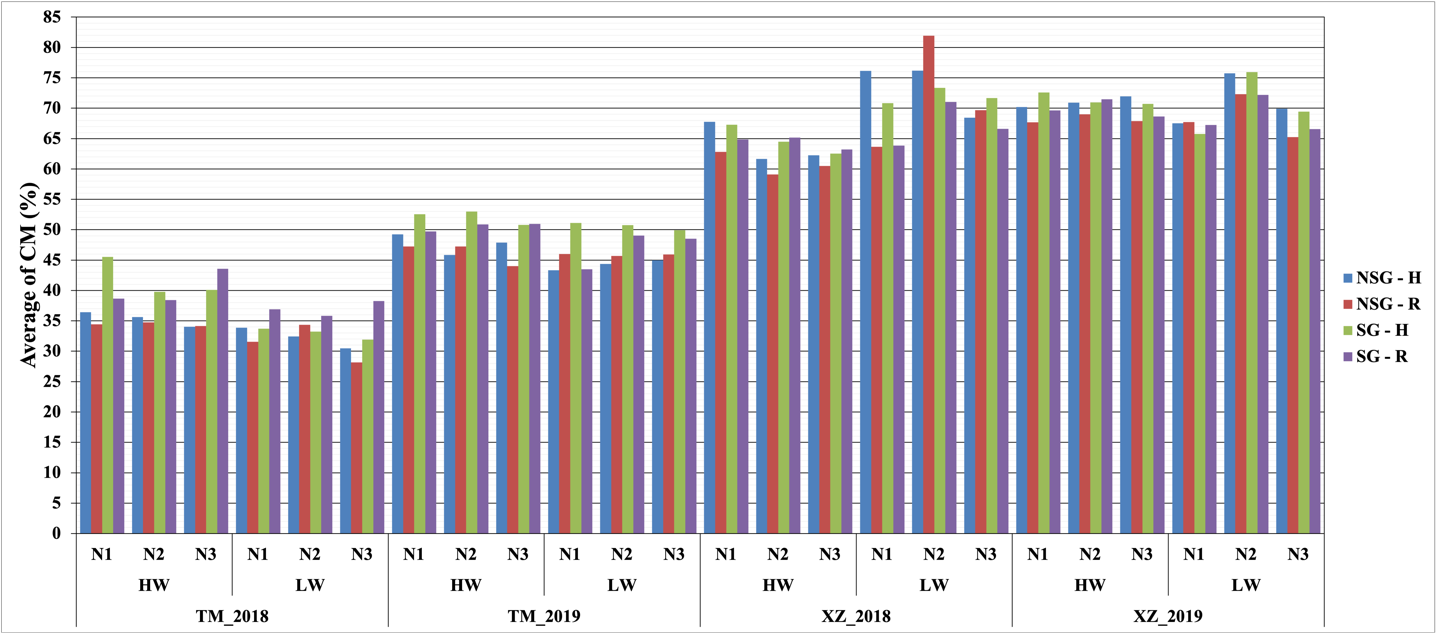


**Supplementary Figure 5.** Average of cob moisture CM (%) within abiotic stresses of water, nitrogen and high plant density for SG and NSG genotypes. (HW: high water irrigation, LW: low water irrigation, N1, N2 and N3: different nitrogen fertilization levels, H: High plant density, R: reduced plant density). Environments (TM_2018, TM_2019, XZ_2018, XZ_2019)
